# Modeling of BN-Doped Carbon Nanotube as High-Performance Thermoelectric Materials

**DOI:** 10.3390/nano12234343

**Published:** 2022-12-06

**Authors:** Naiara L. Marana, Julio R. Sambrano, Silvia Casassa

**Affiliations:** 1 Theoretical Group of Chemistry, Chemistry Department, Torino University, 10125 Torino, Italy; 2 Modeling and Molecular Simulations Group, São Paulo State University, UNESP, Bauru 15385-000, SP, Brazil

**Keywords:** multiwall nanotubes, thermoelectrics, BN doping, DFT

## Abstract

Ternary BNC nanotubes were modeled and characterized through a periodic density functional theory approach with the aim of investigating the influence on the structural, electronic, mechanical, and transport properties of the quantity and pattern of doping. The main energy band gap is easily tunable as a function of the BN percentage, the mechanical stability is generally preserved, and an interesting piezoelectric character emerges in the BNC structures. Moreover, C@(BN)_1−x_C_x_ double-wall presents promising values of the thermoelectric coefficients due to the combined lowering of the thermal conductivity and increase of charge carriers. Computed results are in qualitative agreement with the little experimental evidence and therefore can provide insights on an atomic scale of the real samples and direct the synthesis towards increasingly performing hybrid nanomaterials.

## 1. Introduction

Nanotubes are one of the most interesting nanostructures due to their cylindrical shape, which confers certain properties and opens different possibilities for applications. The properties achieved by the one-dimensional nanostructure (1D) may differ from those of the corresponding two- (2D) and three-dimensional (3D). A classic example is carbon nanotubes (CNT), which have two electronic behaviors, metallic and semiconducting [1], depending on the chirality and the diameter of the nanotubes, whereas graphite (the most stable 3D structure of carbon) and graphene (the surface that generates the nanotubes) are always metallic [2].

Many theoretical and experimental studies have reported the technological applications of this new class of carbon structures, such as gas sensors, voltammetry, enzymatic biosensors, electronic devices and interconnects, field emission devices, electrochemical devices, such as supercapacitors and batteries, nanoscale sensors, electromechanical actuators, separation membranes, filled polymer composites, and many other possible applications [3,4]. Furthermore, studies have shown that single- (SWCNT) and multiwall (MWCNT) carbon nanotubes can resist large deformations and recover elastically, which is not observed for graphite or graphene structures. Finally, Avery [5] and Hung [6], among many others, reported that pure CNTs have potential as thermoelectric materials, which adds another application to the extensive list of carbon nanotube technology exploitation.

As a result of these exceptional properties and multiple applications, new nanotubes of different materials have begun to be synthesized and studied, among which is boron nitride (BN). Its structure is similar to the pristine structure of carbon nanotubes, but unlike CNTs, BN nanotubes (BNNT) have a band gap of around 5.50 eV [7], regardless of the chirality [8]. Due to the structural similarities, many studies have been carried out joining these two materials, forming BNC—carbon boron nitride—structures. By changing the BN concentration on the ternary structures, (BN)_1−x_C_x_, the band gap can be manipulated, and this may pave the way for the production of compounds having specific electronic behavior between that of graphite and boron nitride, making it possible to tune their electronic properties for the needs of specific applications. Besides that, studies have highlighted that BNC structures are considered super-hard materials due to their mechanical properties [9,10], which reinforces their technological appeal. 

Despite the many studies related to investigating the properties of BNC structures [11,12,13,14], only a few are related to nanotubes. Kim and colleagues [15] performed a local Density Functional optimization of BNC double-wall nanotubes with different concentrations of BN and compared their models with the XPS results. They found, in good agreement with experimental findings, that the band gap can be reduced by structural defects along the internal wall up to the value of 1.6 eV. Chiang and colleagues [16] synthesized doped CNT nanotubes with different amounts of BN, forming core–shell heterojunctions, and studied their thermoelectric properties. They measured a significant improvement in the Seebeck coefficient when the percentage of BN reached about 6% and concluded that the ternary hybrid core–shell, C@BCN, has better thermoelectric performance than the pure CNT or BN ones. Sandonas and colleagues [17] simulated BNC-(6,6) single-wall nanotubes using different patterns of doping and BN concentrations and computed for the helical pattern at 50% a figure of merit up to 2.3 times higher than the corresponding values for a CNT at 300 K.

It is evident that in the phase of design and characterization of new materials to improve specific features, theoretical modeling can provide valuable information on the relationships between properties and structure at the atomic scale. In this sense, we propose a complete Density Functional Theory characterization of C@(BN)_1−x_C_x_ zigzag double-wall nanotubes in order to investigate the effect of the quantity and morphology of the walls on their structural, electronic, mechanical, and transport properties. This study allows us to outline general trends and suggest lines of research toward the engineering of realistic and promising materials to be used as thermoelectric devices.

The manuscript is organized as follows: in the next section, methodologies (equations and theoretical background) and computational setup (DFT functionals, basis set, convergence criteria, and tolerances) are detailed. Then, pure BN and C double-wall structures are analyzed, and, subsequently, different hybrid nanotubes are modeled, by varying the percentage *x* of BN and its doping pattern. Thermoelectric quantities are finally calculated and compared with those of the single-wall structures ((BN)_1−x_C_x_) and pristine materials. Summary and general conclusions are given at the end.

## 2. Materials and Methods

Periodic computational simulations using Density Functional Theory (DFT) were carried out with the CRYSTAL23 program [18], a quantum-mechanic periodic code that solves the Schrödinger equation by expanding the wave function in a basis set of Gaussian-type orbitals, centered in the atomic positions. The hybrid version of the GGA Perdew–Burke–Ernzerhof [19] functional with 25% of the exact Hartree–Fock exchange [20] (PBE0) is adopted along with its empirical corrections to include long-range dispersion effects (PBE0-D3) as proposed by Grimme [21] and modified for solid-state systems [22].

The all-electron 6-21G* basis set is used for carbon [23], boron [24], and nitrogen [23] atoms. The computational setup, i.e., functional, basis set, integrals cut-off, which determines the accuracy of the methodology, is based on the results of previously published research [25,26].

In particular, the shrinking factor for both the diagonalization of the Fock matrix and the calculation of the energy is set to 4, corresponding to three independent **k**-points in the irreducible part of the Brillouin zone. Geometry optimization is performed using analytical gradients with respect to atomic coordinates and unit cell parameters within a quasi-Newtonian scheme combined with the Broyden–Fletcher–Goldfarb–Shanno procedure for the updating of the Hessian [27,28,29]. For each equilibrium structure, the full set of vibrational frequencies in Γ is obtained, within the harmonic approximation, by diagonalizing the mass-weighted Hessian matrix. These quantities are used to evaluate the zero-point energy (ZPE) and the thermal contributions to the vibrational energy and entropy in order to estimate the Gibbs free energy:

(1) $$G\left(T\right)=E_{el}+E_{ZPE}+E_{vib}+PV-T\;S_{vib}$$

Elastic (*c11*) and piezoelectric (*e11*) constants along the periodic direction of the nanotube (*x*-axis) were calculated with CRYSTAL by computing the polarization via the Berry phase approach. This methodology has already been tested on similar materials and provided good general agreement with both experimental and theoretical results [30]. The thermodynamic stability of these structures was accounted for by performing a 3 ps Born–Oppenheimer molecular dynamics simulation on the C@CBN model, within the canonical ensemble at *T* = 300 K, with a beta version of the CRYSTAL code [31,32].

For the evaluation of the thermoelectric quantities, i.e., the Seebeck coefficient (*S =* Δ*V/*Δ*T*), the electron conductivity (*σ*), and the power factor (*PF = S*^2^*σ*), a denser net was adopted in the reciprocal space, containing 200 **k**-points along the periodic direction. These properties were calculated using the semi-classical Boltzmann transport equation theory [33] (BTE), as implemented in the CRYSTAL code, within the frozen band approximation, assuming the energy relaxation time for carriers (τ) a constant parameter [34]. The latter was set to 12 *fs*, according to our previous work on multiwall carbon nanotubes (MWCNT) [30]. For the total thermal conductivity (***κ***) necessary to provide a tentative estimation of the figure of merit (*ZT = S*^2^*σ T/**κ***), values in the range from 1500–2500 W K^−1^ m^−1^ were used as a function of the BN doping percentage, in agreement with the study of Sandonas et al. [17] on BNC hetero-nanotubes. These quantities can also be derived by decreasing the reference experimental value for the carbon multiwall [35], 3000 W K^−1^ m^−1^, for a quantity that takes into account the effects of the BN doping on the thermal conductivity, on the basis of the measures performed by Chang et al. [36] on mats and independently by Pop and co-workers on single-wall nanotubes [37].

In order to highlight the modifications on the electronic and geometric structures introduced by the BN dopant on carbon double-wall nanotubes, as well as their repercussions on the transport properties, we turned our attention to the few experimental studies on the thermoelectric performance of carbon-based hybrid nanostructures.

We therefore used as a starting point the C@BNC nanotubes synthesized and characterized by Chiang et al. [16] and designed a set of double-wall structures with different doping concentrations and patterns. Such models represent the possible atomic-scale engineering of their promising samples.

**Figure 1:**
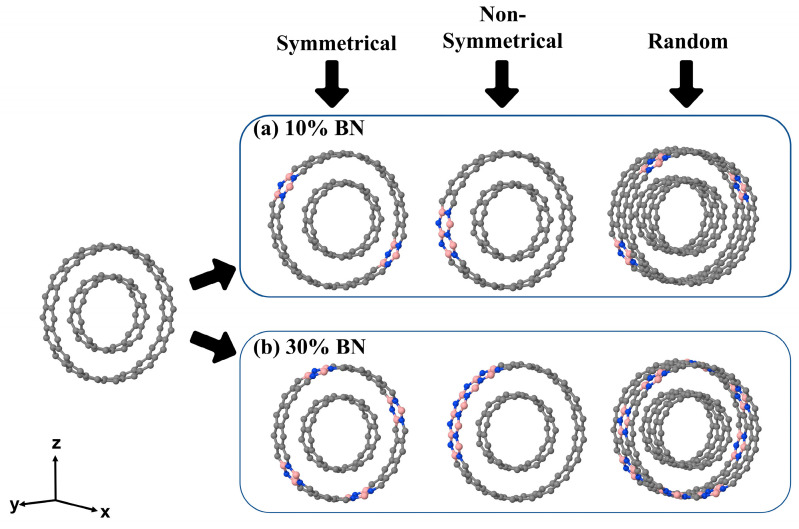
Unit cell of the double-wall zigzag (11,0)@(20,0) carbon nanotube. The pristine model in the left panel (44@80 atoms) and the corresponding BN-doped structures, C@(BN)_1−x_C_x_, are shown in the upper right, x = 0.10, and lower, x = 0.30, panels, respectively, for the three different patterns. In the case of the *random* pattern, to preserve periodicity along x, the cell must be double. Color of the atoms: gray-carbon, blue-nitrogen and pink-boron.

The reference double-wall carbon C@C and BN@BN nanotubes were modeled according to a new methodology recently implemented in the CRYSTAL program, successfully applied to the study of multiwall nanotubes [30], and soon available in the new public version of the code. In particular, the zigzag double-wall nanotubes were generated by rolling up a previously optimized sheet of graphene and the graphene-like (0001) BN surface, respectively. Among different possibilities, the double-wall (11,0)@(20,0) nanostructure, already characterized in the case of carbon nanotubes [30], turns out to be the one with the most favorable energy balance, also in the case of the BN@BN (see Supplementary Materials Table S2). Their unit cell, shown in Figure 1 (and Supplementary Materials Figure S1, in the case of BN@BN), contains 44 and 80 carbon atoms in the inner (core) and outer (shell) wall, respectively, for a total of 124 atoms. Starting from the (11,0)@(20,0) carbon structure, a set of BN-doped double-wall hetero-nanotubes were created, C@(BN)_1−x_C_x_, by replacing some atoms of the outer wall with BN in a concentration 0.10 < x < 0.30. These models are shown in the right panels of Figure 1 and information on their unit cells is summarized in Table S1. Here and in the following, x represents the percentage of BN atoms out of the total number of carbon atoms of the outer wall (i.e., 80). Then, in accordance with the line of research opened by Sandonas et al. [17] on single-wall nanotubes, we have designed three different doping patterns to investigate the combined effects of the amount and morphology of BN. Referring to Figure 1, these structures were named: (i) *symmetrical*, (ii) *non-symmetrical,* and (iii) *random*, according to the arrangement of the hexagons formed by the BN heteroatoms along the periodic direction. Interestingly, the structural unity of cyclohexane is always preserved. 

**Figure 2:**
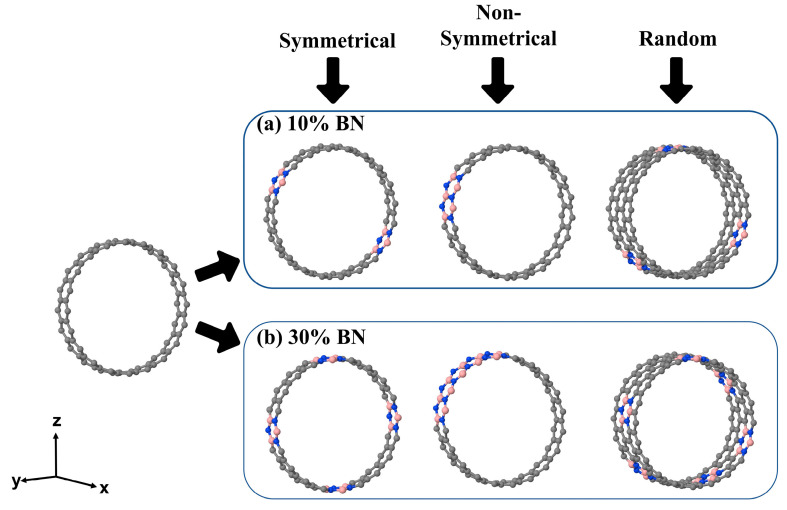
Unit cell of the single-wall zigazag (20,0) carbon nanotubes. The pristine model in the left panel (80 atoms) and the corresponding BN-doped structures, (BN)_1−x_C_x_, are shown in the upper right, x = 0.10, and lower, x = 0.30, panels, respectively, for the three different patterns. As for the double-wall, in the case of the *random* pattern, to preserve periodicity along x the cell must be double. Color of the atoms: gray-carbon, blue-nitrogen and pink-boron.

For comparison, single-wall nanotubes (20,0) (BN)_1−x_C_x_, were also generated and characterized, with x covering the same concentration range as in the outer wall of the corresponding double-wall system (see Figure 2). 

The energy of the double-wall nanotubes was evaluated by means of three different quantities. The formation energy *Eform* is defined as follows:

(2) $$Eform=E_{nw}/n_{nw}-E_{n-layers}/n_{n-layers}$$

where $E_{nw}$ and $E_{n-layers}$ are the total energies of the fully optimized (cell parameters and volume) single- (1 W) and double-wall (2 W) nanotubes and n-layered slab (one layer for the single and two layers for the double-wall nanotubes), respectively, and *n_x_* is the number of atoms in each reference cell. The inter-wall interaction *Eiw* provides an estimate of any interaction between the tubes:

(3) $$Eiw=1/n_{2w}\left(E_{2w}-\sum_{SW}E_{SW}\right)$$

where *E_SW_* are the energies of the two optimized isolated single-wall structures. 

The energy *Emix*, calculated considering the formation of the heterostructure C@BNC in a hypothetical equilibrium reaction with the pristine C@C, with a bulk of boron nitride and graphite as reservoirs for the BN and C atoms, respectively:

(4) $$Emix=E_{CBN}+E_{bulk-C}-E_{nW-C}-E_{bulk-BN}$$

where *nW* corresponds to a single- or double-wall carbon nanotube and $E_{bulk}$ is the energy of BN ($E_{bulk-BN}$) and graphite ($E_{bulk-C}$). *Emix* provides a possible estimate of the relative stability of the doped double-wall nanotube with respect to the pristine one and therefore of the ease with which the mixed compounds could be obtained, working in excess of BN.

It is important to underscore that at any finite temperature, the properties of the C@BNC systems can differ considerably from those calculated at the DFT level, i.e., at *T* = 0 K. We have partly taken into account the temperature in the estimation of the Gibbs free energy of formation, evaluated according to Equation (4), and in the calculation of the transport properties where, within the frozen band approximation, the state’s occupation varies according to the Fermi distribution.

## 3. Results and Discussions

### 3.1. Structural Properties of C@(BN)_1−x_C_x_

As regards the structural characterization of carbon double-wall nanotubes, DW-CNT, a reference can be made to a previous article [30]. Instead, a preliminary study on BN@BN nanotubes was conducted to acquire information on their structural and electronic properties. As in the case of DW-CNT, DW-BN has bond lengths and angles close to those of the precursor (0001) two-layer surface, and this indicates that the wrapping of the layers takes place without significant structural stresses and distortions (see Tables S2–S4). The *Egap* for BN, at the PBE0 level, is that of a semiconductor, ranging from 3.59 to 4.12 eV, slightly less than that experimentally measured [38]. Interestingly enough, the double-wall BN, with the lower energy evaluated according to Equation (2), has the same structure that was computed for C@C, characterized by an internal/core diameter and an external/shell diameter of 8.95 and 16.15 Å, respectively, an inter-wall equilibrium distance of 3.66 Å, and 124 atoms in the reference periodic unit. The possible fine-tuning of the gap and the structural similarity with the carbon lattice are precisely the characteristics that make doping with BN particularly interesting. For the sake of completeness, the energetic and structural information of the reference (11,0)@(20,0) C@C and BN@BN double-wall systems are reported in Table 1.

Then, we started to model the double-wall C@(BN)_1−x_C_x_ heterostructures. After a preliminary screening, based on the computed values of *Emix* and *Egap*, we concentrated our attention on two percentages of doping: 10% and 30% (out of the 80 atoms of the outer wall) corresponding to the 6% and 20% on the entire DW. The structural properties of these ternary double-walls are shown in Table 2, together with their main band gap energy and inter-wall energies *Eiw*, which is a measure of any interaction between the core and the shell walls. Also in Table 2, the same quantities are reported for the corresponding single-wall (BN)_1−x_C_x_ nanostructures.

As can be seen in Table 2, the C@(BN)_1−x_C_x_ nanotubes have diameters, C–C and B–N bond lengths, and inter-wall distances very similar to those of the pristine C@C double-wall. This indicates that BN doping does not introduce appreciable structural distortions and can occur within a wide range of concentrations. The values of *Emix* are in fact very low and decrease as the number of joints between the carbon lattice and the doping atoms decreases, the *non-symmetrical* structures being the ones forming at the lowest energy cost. This phenomenon has already been observed and well documented in the literature. Many theoretical studies [12,39] have analyzed the influence of the so-called nanodomains, i.e., small BN (or C) clusters dispersed into pure C (or BN) structures, on the formation energy of BNC hybrid compounds. The presence of nanodomains increases the number of interfaces, each of which is associated with a discrete energy cost. Indeed, the formation of boundaries leads to a reduction in the cohesion energies, since the C–C and B–N bonds are more effective than the mixed C–B and C–N ones, and an enhancement of the structural strain. These effects do not prevent the formation of ternary structures but, as the BN concentration increases, tend to favor the formation of homogeneous zones and minimize the extension of the interfaces. 

Furthermore, Kah et al. [14] found that not only does the substitution pattern affect the stability, but the energy difference between the C–B and C–N interactions can also determine the type of boundaries that form. If, in the case of h-BNC layers, the C–B edges make the sheets less stable, in nanotubes this effect is yet to be explored and understood.

In this context, we can proceed to read and interpret our results. At low concentrations, *random* models have the maximum energy of formation and the maximum attractive interaction, as confirmed by the equilibrium distance between the walls, which is the shortest. Both for SW and DW, the *non-symmetrical* substitution pattern is formed with the minimum expenditure of energy. Such an effect can be attributed to the lower amount of B–C and N–C bonds and the minimum number of C/BN joints. In agreement with the studies just mentioned [14,39,40], for a doping concentration of 30%, the number of boundary B–C and N–C bonds per unit cell is 12, 8, and 2 in the *random*, *symmetrical*, and *non-symmetrical* structures, respectively, in excellent agreement with the decreasing trend calculated for *Emix*. Then, it is interesting to compare the values of *Eiw* of the C@(BN)_1−x_C_x_ ternary structures with those of C@C which demonstrate the increase in attractive, and therefore stabilizing, interactions between the two walls when BN atoms are present. This effect is magnified when dispersive interactions are taken into account by means of the D3 a posteriori correction (for the C@(BN)_0.10_C_0.90_ *non-symmetrical*, the *Emix* = −0.10 eV and the *Eiw* = −0.032 eV). Consequently, DW hybrids benefit from an extra stabilizing contribution compared to SW, due to the favorable interaction between the walls.

To estimate the relative stability of the proposed structure at room temperature, the Gibbs free energies evaluated according to Equation (4), *Gmix*, were calculated for the symmetrical and non-symmetrical double-wall with 10% and 30% doping. Values are +8.42 eV (C@(BN)_0.30_C_0.70_ *non-symmetrical*), +9.93 eV (C@(BN)_0.10_C_0.90_ *non-symmetrical*), +15.34 eV (C@(BN)_0.10_C_0.90_ *symmetrical*), and +18.32 eV (C@(BN)_0.30_C_0.70_ *symmetrical*) and reproduce the trend obtained considering only the electronic contribution, i.e., *Emix*, confirming the C@(BN)_0.30_C_0.70_ *non-symmetrical* as the most favored structure from the thermodynamic point of view.

As regards the mechanical properties, carbon structures in general have good elastic behavior and are considered resistant materials. It was already demonstrated by Hultman and co-authors that the presence of BN, but also S atoms, contributes to increasing the rigidity of carbon thin films [10,40]. Carbon nanotubes were also found to exhibit excellent mechanical properties, very high resistance, and high elastic constants [30], and recent studies [41,42,43,44] showed that CBN materials retain elastic constants higher than that of pure BN ones. Our results are in perfect agreement with the literature data: (i) the Born criteria, namely *c11* > 0, is satisfied for all the structures, and as the amount of BN doping increases, the elastic constants of the C@(BN)_1−x_C_x_ ternaries slightly decrease. The same predictable behavior is calculated for the SW nanotubes. Nevertheless, as expected, the computed elastic constants of DW nanotubes are ~1.5 times greater than that of the SW structures, and a dopant concentration of approximately 30% preserves the excellent mechanical stability of the pristine carbon double-wall. This is in accordance with what can be expected based on the number of atoms per cell, which in the case of DW is about 1.5 times greater than that of SW. As regards the influence of the doping pattern, the *random* structures, which locally break the translational symmetry, are those with the minor value of *c11*.

Regarding the piezoelectric properties, our calculations show that zigzag carbon nanotubes do not exhibit piezoelectricity. Therefore, the piezoelectricity values obtained for the hybrid structures and reported in Table 1 are evidently due to doping with BN. The structures (BN)_0.30_C_0.70_ models, both for SW and DW, are those with the highest values of *e11*, even higher than that of pure BN, and with the same concentration, the *non-symmetrical* pattern is the one with the greatest piezoelectric character.

Finally, NVT molecular dynamics calculations at T = 300K were performed; as can be seen from Figure S3, for the *non-symmetrical* system with x = 30%, the MW structure remains unchanged, and the total energy oscillates around the value of equilibrium, confirming substantial thermal stability at room temperature.

### 3.2. One-Electron Properties

BN nanotubes, originally proposed by Rubio et al. (1994) [45], possess a geometrical structure very similar to their carbon counterpart but exhibit radically different electronic properties. Independent of the nanotube diameter and chirality, no metallic BN nanotubes are observed. In the engineering of high-performance thermoelectric materials, the goal is to overcome the metallic character of carbon nanotubes, reduce their thermal conductance, and continue to exploit their very high electrical conductivity. Band gap manipulation uses doping as a way to modify the material gap to one that is more favorable for a given application. Therefore, the different electronic characteristics of C and BN have been exploited in the synthesis of hybrid carbon nanostructures based on molecular doping with BN. 

Studies on B- and N-substitutionally-doped carbon nanotubes have shown that their electronic properties strongly depend on both the composition and atomic configuration [15,46,47]. In particular, theoretical calculation and experimental measurements predict that BNC single- and multiwall are always semiconductors and possess a band gap that is adjustable by varying the chemical composition and atomic configuration [12,17]. The same behavior was found in multiwall heterostructures [16] and is confirmed by our calculations.

The values of *Egap,* reported in Table 2, show that the C@(BN)_1−x_C_x_ and (BN)_1−x_C_x_ become semiconductors and that there is an almost linear correlation between the percentage of BN and the opening of the energy gap. 

**Figure 3:**
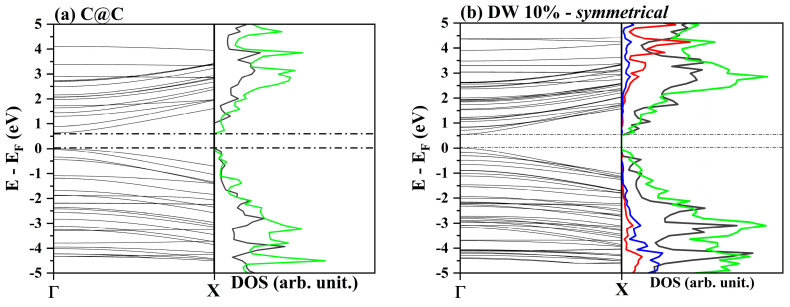

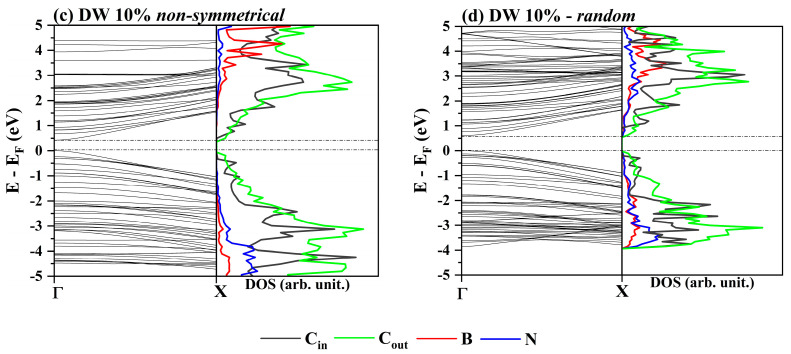
Band structures and DOS for zigzag nanotubes: a comparison between double-wall C@C (**a**) and the 10% doped C@(BN)_0.10_C_0.90_ with different patterns, i.e., *symmetrical* (**b**), *non–symmetrical* (**c**), and *random* (**d**). Carbon atoms of the internal and external walls are represented by gray and green lines, respectively, while boron and nitrogen are represented by red and blue lines, respectively.

In Figure 3, the band structures and density of states (DOS) of the double-wall nanotubes doped with 10% BN are shown. The results of C@(BN)_0.10_C_0.90_ were compared with the C@C, shown in Figure 3a. Then, it is possible to affirm that (i) the low-energy electronic transitions occur between states which mainly belong to the carbon atoms and (ii) the greatest contribution to the lower part of the conduction bands comes from the atoms of the external wall and are due to *p_y_* and *p_z_* orbitals, which are much less involved in the C–C bonds along the periodic x-direction. This observation leads us to conclude, in agreement with Chiang and colleagues [16], that to modify the conduction and transport properties of these materials, it is appropriate to work on the atomic structure of the outer shell. Finally, it is worth noting that in the *random* model, the valence states in the region around −4.5 eV, which are characteristic of the periodic rows of BN, are missing. 

### 3.3. Thermoelectric Properties

In summary, the doping of carbon nanotubes with BN increases the thermoelectric performance of pristine materials for at least two reasons: the tuning of the band gap into optimal values and the lowering of the thermal conductivity, as documented by, among others, Zettl and colleagues [48]. To these properties is added remarkable stability to these materials, and our *Emix*, computed for the SW and DW, goes exactly in this direction.

In their pioneering work, Chiang et al. [16] indicated the double-wall systems, made of metallic carbon cores and semiconducting BNC shells, C@CBN, as the ones with the best performance, due to a good compromise between conductivity and Seebeck coefficient. About this, the computed density of states confirms the influence of the outer wall composition in shaping the density around *Efermi.*

By varying the BN content of the external wall, Chiang et al. measured the highest Seebeck coefficient, 22.8 µV/K, corresponding to a power factor of 0.64 µW/mK^2^, for the sample containing 10% of BN atoms in the outer wall, at 300 K (corresponding to the 6% on the total). In the range of 10–30% (6%–20%), both the Seebeck coefficient and *PF* showed optimum values, and only when the percentage of doping increased over 40% (30%) did the thermoelectric performances start to decrease monotonously. Finally, they performed a temperature-dependent characterization of the sample at 10% (6%) in BN, concluding that in the range 300–600 K, as the electrical conductivity decreased, both *S* and *PF* slightly increased (23 → 28, 0.6 → 1.4) [15]. 

If Chiang et al. [16] pointed out with excellent precision the unique relationship between thermoelectric properties and BCN doping levels, the atomic-scale characterization of their samples, for obvious reasons, was somewhat simple. As such, the effect of different doping patterns on the thermopower of C@BNC systems is still unclear. At this point, theoretical models can help define a possible relationship between the geometric and electronic structure of these materials and their properties as thermoelectrics.

**Figure 4:**
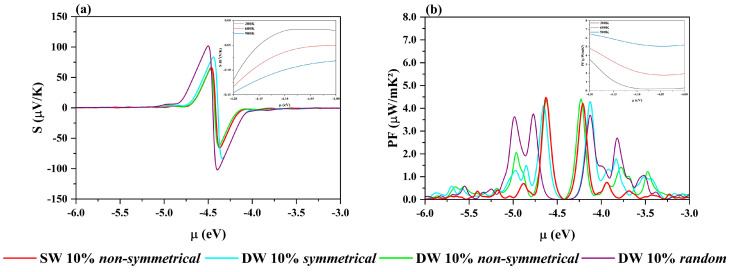
(**a**) Seebeck coefficient (µV/K) and (**b**) power factor (μW/mK^2^) of single*–* (in red) and double–wall nanotubes with 10% BN with different doping patterns: *symmetrical* in cyan, *non–symmetrical* in green, and *random* in purple. Inset: the *non–symmetrical* C@(BN)_0.10_C_0.90_ at different temperatures for the value of the chemical potential −4.20 eV, corresponding to a carrier concentration of −3.75 10^−22^.

We first computed the transport coefficients for the double-wall hybrid materials with 10% of BN, corresponding to the Chiang sample at 6%. The results, collected in Figure 4, show that the morphology of the outer wall has a strong influence on both Seebeck and the power factor. The *random* pattern, with an *Egap* of 0.67 eV, presents the higher *S*, with a peak value of ~110 µV/K, but has a *PF* compatible with that of a *p-type* semiconductor (purple line). On the other hand, the *non-symmetrical* structure (green line) which presents lower values of Seebeck, shows particularly interesting features. At the maximum value of *PF*, ~4.1 µW/mK^2^, which is also the highest value among all those calculated, the carrier concentration is about −3.7510^−22^ and *S* about 40 µV/K. Then at this carrier concentration, as the temperature rises, passing from 300 to 600 K, the *PF* doubles and the Seebeck increases, as shown in the two insets of Figure 4. 

The qualitative agreement with the data measured by Chiang et al. is impressive [16]. All the experimental trends are reproduced, and the overestimation of the quantities by the calculated values is probably due to (i) the approximations of the method and (ii) the models which, while simulating local disordered patterns, must possess a long-range order. 

**Figure 5:**
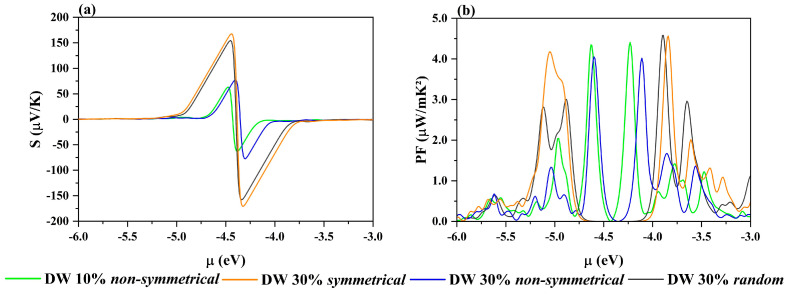
(**a**) Seebeck coefficient (µV/K) and (**b**) power factor (µW/mK^2^) for 30% BN-doped DW structures, C@(BN)_0.3_C_0.7_. *Symmetrical*: orange; *random*: gray; *non–symmetrical:* blue. For the sake of comparison, the C@(BN)_0.10_C_0.90_ *non–symmetrical* is also added, in green.

By increasing the BN percentage, 30% of doping is reached; again, the *random* and *symmetrical* C@(BN)_0.30_C_0.70_ structures present impressive peak values for the Seebeck coefficients, as reported in the (a) panel of Figure 5. However, their power factor is lower than that of the *non-symmetrical* model (blue line), which is once again the most interesting as far as transport properties are concerned. Its *PF* is only slightly lower than that of the corresponding 10% doped model (green line). 

As the BN content exceeds 30%, the band gap widens, and the material loses interest as regards its possible use in thermoelectric applications. For completeness, both Seebeck and the power factor for all systems can be found in Supplementary Materials Figure S2. 

Finally, with the aim of comparing the thermoelectric efficiency between single- and double-wall structures, we performed the same analysis on the (BN)_1−x_C_x_ models (Supplementary Materials Figure S2). In Figure 4, the thermoelectric features of the best SW, the *non-symmetrical* at 10%, are superimposed on those of the DW. Despite the extremely high values of the Seebeck coefficients of the single-wall models, when the power factors are considered, the double-wall structures perform better. 

To complete the comparison, we used reliable experimental values for thermal conductivity to estimate *ZT* for the *non-symmetrical* SW and DW with 10% of BN (***κ_SW_*** = 3000 and ***κ_DW_ =*** 2500 W K^−1^ m^−1^). The calculated values, 0.41 and 0.43, respectively, for the single- and double-wall models, are an order of magnitude higher than those obtained so far on experimental samples, but once again the comparison is in favor of the double-wall structure. 

## 4. Conclusions

DFT was applied to the study of C@(BN)_1−x_C_x_ and (BN)_1−x_C_x_ ternary nanotubes, with x = 0.10 and 0.30 and different doping patterns. The analyzed nanotubes were structurally and energetically stable according to the formation and inter-wall energies. The predictions on the energy gap are in agreement with the literature results for different BNC ternary materials. 

We can therefore conclude that the double-wall BN-doped carbon nanotubes can well manipulate C@C material into good thermoelectrics thanks to the opening of the energy gap. Furthermore, C@(BN)_1−x_C_x_ are more appealing compared to single-wall nanotubes due to their mechanical stability and lower thermal conductivity, while they maintain high electrical conductivity and high values of the carrier concentration.

According to our results, the best performance can be achieved with a doping percentage of about 10% of BN in the external wall, corresponding to a total amount of BN of around 6%. Furthermore, from our modeling on the atomic scale, it was possible to deduce that the structures in which the heteroatoms of BN fit into the outer wall and form a homogeneous pattern, i.e., the *non-symmetrical* models, are those that have the best values of formation energy, mechanical stability, piezoelectric character, and thermal power.

This study highlights the possibility of applying nanotubes as thermoelectric materials and suggests synthesis paths toward the engineering of increasingly high-performing nanomaterials.

## Figures and Tables

**Table 1:** Structural properties (in Å) and energetics (in eV) at the PBE0 level of the reference systems: double-wall (11,0)@(20,0) zigzag carbon and BN nanotubes and (20,0) single-wall nanotubes. Bond length (C–C, B–N, C–B, and C–N), inter-wall distance (*diw*), internal and external diameters (*Dint* and *Dext*), formation and inter-wall energies (*Eform*, *Eiw*), band gap energy (*Egap*), elastic (*c11*, Hartree), and piezoelectric constants (|*e11*|, |e|*Bohr).

	C–C	B–N	*diw*	*Dint*	*Dext*	*Eform*	*Eiw*	*Egap*	*c11*	*e11*
BN@BN	-	1.45	3.45	9.07	15.98	0.018	−0.009	5.84	210.11	67.94
C@C	1.43	-	3.50	8.71	15.71	0.067	−0.002	0.64	261.75	0
C	1.42	-	-	-	15.72	0.043	-	0.67	174.04	0
BN	-	1.45	-	-	16.16	0.010	-	6.74	136.30	44.08

**Table 2:** Structural properties (in Å) and energetics (in eV) at the PBE0 level of single (20,0) and double-wall (11,0)@(20,0) zigzag carbon nanotubes with different BN doping percentages and patterns. Bond length (C–C, B–N, C–B, and C–N), inter-wall distance (*diw*), internal and external diameters (Dint and Dext), formation and inter-wall energies per atom (*Emix, Eiw*), band gap energy (*Egap*), elastic (*c11*, Hartree), and piezoelectric constants (|*e11*|, |e|*Bohr).

	C–C	B–N	C–B	C–N	*diw*	*Dint*	*Dext*	*Emix*	*Eiw*	*Egap*	*c11*	*|e11|*
*Symmetrical*												
10%SW	1.42	1.46	1.51	1.40	-	-	16.70	0.045	-	0.57	165.03	88.77
30%SW	1.43	1.45	1.50	1.42	-	-	15.84	0.087	-	1.44	157.57	96.61
10%DW	1.43	1.45	1.51	1.39	3.35	8.94	15.63	0.028	−0.081	0.55	255.74	74.58
30%DW	1.43	1.46	1.51	1.40	3.53	8.72	15.77	0.057	−0.074	1.07	251.62	95.27
*Non-symmetrical*												
10%SW	1.42	1.45	1.52	1.40	-	-	16.39	0.021	-	0.45	161.80	89.83
30%SW	1.43	1.45	1.52	1.40	-	-	16.33	0.016	-	0.55	156.69	104.61
10%DW	1.43	1.45	1.52	1.40	3.44	8.70	15.58	0.013	−0.087	0.43	256.21	92.23
30%DW	1.43	1.47	1.52	1.39	3.36	8.93	15.65	0.011	−0.085	0.52	250.82	100.94
*Random*												
10%SW	1.43	1.45	1.49	1.40	-	-	15.78	0.049	-	0.81	164.43	76.48
30%SW	1.43	1.45	1.49	1.40	-	-	15.74	0.139	-	1.45	156.87	102.62
10%DW	1.43	1.45	1.49	1.39	3.47	8.71	15.65	0.029	−0.086	0.67	247.35	81.55
30%DW	1.43	1.45	1.49	1.39	3.52	8.71	15.75	0.088	−0.091	0.99	244.41	100.85

## Data Availability

Not applicable.

## Supplementary Materials

The following supporting information can be downloaded at: https://www.mdpi.com/article/10.3390/nano12234343/s1, Table S1: Information on the unit cell for each model system, is shown in Figure 1 and Figure 2 of the manuscript. Total number of atoms, $n_{\mathrm{tot}}$, carbon atoms in the inner wall (core), $n_{\mathrm{core}}^{C}$, carbon atoms in the outer wall (shell), $n_{\mathrm{shell}}^{C}$, and boron and nitrogen atoms in the outer wall (shell), $n_{\mathrm{shell}}^{\mathrm{BN}}$ for the double and single-wall systems and the different doping patterns; Table S2: Number of atoms (*nat*), bond length (B–N, in Å), inter-wall distance (*diw*, in Å), bond angle (B-$\hat{N}$-B, in degrees), internal and external diameters (*Dint* and *Dext*, respectively, in Å), formation (*Eform*, in eV), inter-wall (*Eiw*, in eV), and band gap energies (*Egap*, in eV) for double-wall boron nitride nanotubes, at the PBE0 level. Due to its key role, information on the structure of the carbon nanotube (11,0)@(20,0) are reported; Table S3: Bond length (B–N in Å) and angle (B–$\hat{N}$–B in degrees), band gap energy (Egap in eV), elastic (c11, Hartree), and piezoelectric (|e11|, |e|*Bohr) constants of BN bulk, surface, and single-walled nanotubes at the PBE0 level; Table S4: Bond length (C–C in Å) and angle (C–$\hat{C}$–C in degrees), band gap energy (*Egap* in eV), elastic (*c11*, Hartree), and piezoelectric (*e11*, |e|*Bohr) constants of C bulk, surface, and single-walled nanotubes; Figure S1: Unit cell of BN@BN double-walled nanotubes whose structural and energetic properties are reported in Table S2; Figure S2: (a) Seebeck coefficient (mV/K) and (b) power factor (mW/mK^2^) of (BN)_1−x_C_x_ single-wall nanotubes; Figure S3: Molecular dynamics simulation of C@(BN)_0.10_C_0.90_ *non-symmetrical* within the canonical ensemble at *T* = 300 K (NVT).
