# Supplementary material for: Modeling of BN-Doped Carbon Nanotube as High-Performance Thermoelectric Materials
Source: Nanomaterials (Basel). 2022 Dec 6;12(23):4343. doi: 10.3390/nano12234343 (PMC9737904; doi:10.3390/nano12234343)
Supplement: Supplementary file 1 [file nanomaterials-12-04343-s001.zip › nanomaterials-2081921-supplementary.pdf]

Modeling of BN-doped carbon nanotube as high-performance thermoelectric materials

Naiara L Marana[2], Julio R Sambrano[1], and Silvia Casassa[2,*]

[1]*Modeling and Molecular Simulations Group, São Paulo State University, UNESP, Bauru, SP, Brazil*

[2]*Theoretical Group of Chemistry, Chemistry Department, Torino University, Torino, Italy*

Supplementary Material

Table S1: Information on the unit cell for each model system, is shown in Figures 1 and 2 of the manuscript. Total number of atoms, $\boldsymbol{n_{tot}}$, carbon atoms in the inner wall (core), $\boldsymbol{n^{C}_{core}}$, carbon atoms in the outer wall (shell), $\boldsymbol{n^{C}_{shell}}$, and boron and nitrogen atoms in the outer wall (shell), $\boldsymbol{n^{BN}_{shell}}$ for the double and single-wall systems and the different doping patterns.

	**Pattern**	n_{tot}	n^{C}_{core}	n^{C}_{shell}	n^{BN}_{shell}
**C@C**	--	124	44	80	--
**C@(BN)$_{0.10}$C$_{0.90}$**	symmetrical	124	44	68	12
**C@(BN)$_{0.10}$C$_{0.90}$**	non-symmetrical	124	44	70	10
**C@(BN)$_{0.10}$C$_{0.90}$**	random	248	88	142	18
**C@(BN)$_{0.30}$C$_{0.70}$**	symmetrical	124	44	56	24
**C@(BN)$_{0.30}$C$_{0.70}$**	non-symmetrical	124	44	58	22
**C@(BN)$_{0.30}$C$_{0.70}$**	random	248	88	112	48
**(BN)$_{0.10}$C$_{0.90}$**	symmetrical	80	--	68	12
**(BN)$_{0.10}$C$_{0.90}$**	non-symmetrical	80	--	70	10
**(BN)$_{0.10}$C$_{0.90}$**	random	80	--	142	18
**(BN)$_{0.30}$C$_{0.70}$**	symmetrical	80	--	56	24
**(BN)$_{0.30}$C$_{0.70}$**	non-symmetrical	80	--	58	22
**(BN)$_{0.30}$C$_{0.70}$**	random	80	--	112	48

Table S2: Number of atoms (*nat*), bond length (B-N, in Å), inter-wall distance (*diw*, in Å), bond angle (B-$\hat{N}$-B, in degrees), internal and external diameters (*Dint* and *Dext*, respectively, in Å), formation (*Eform*, in eV), inter-wall (*Eiw*, in eV), and band gap energies (*Egap*, in eV) for double-wall boron nitride nanotubes, at the PBE0 level. Due to its key role, information on the structure of the carbon nanotube (11,0)@(20,0) are reported.

**BN@BN**	***nat***	***B-N***	***diw***	***B-$\hat{N}$-B***	***Dint***	***Dext***	***Eform***	***Eiw***	***Egap***
**(10,0)@(20,0)**	120	1.46	4.00	119.4	8.18	16.08	-0.28	-0.007	5.63
**(11,0)@(20,0)**	124	1.46	3.66	119.8	8.95	16.15	-0.29	-0.008	5.84
**(12,0)@(20,0)**	128	1.46	3.34	119.8	9.61	16.24	-0.29	-0.007	6.04
**(13,0)@(20,0)**	132	1.45	3.30	120.0	10.53	16.43	-0.27	0.008	6.18
**(15,0)@(20,0)**	140	1.42	2.68	121.6	11.60	17.00	-0.19	0.099	6.23
**C@C**	**n_{at}**	**C-C**	**d_{IW}**	**C-$\hat{C}$-C**	**D_{int}**	**D_{ext}**	E_{form}	E_{IW}	E_{gap}
**(11,0)@(20,0)**	124	1.42	3.50	120	8.71	15.71	0.07	-0.0025	0.64

Table S3: Bond length (B-N in Å) and angle (B-$\hat{N}$-B in degrees), band gap energy (*Egap* in eV), elastic (*c11*, Hartree), and piezoelectric (|*e11*|, |e|*Bohr) constants of BN bulk, surface, and single-walled nanotubes at the PBE0 level.

	**B-N**	**B-$\hat{N}$-B**	**Egap**	**c11**	**|e11|**
**Bulk**	1.46	120.0	7.32	807.91	0.003
**monolayer**	1.46	120.0	8.26	3.64	0.00
**Bilayer**	1.46	120.0	7.75	7.59	0.00
**SW-(11,0)**	1.46	119.5	6.24	73.86	24.48
**SW-(20,0)**	1.46	120.0	6.74	136.30	44.08

Table S4: Bond length (C-C in Å) and angle (C-$\hat{C}$-C in degrees), band gap energy (*Egap* in eV), elastic (*c11*, Hartree), and piezoelectric (*e11*, |e|*Bohr) constants of C bulk, surface, and single-walled nanotubes

	**C-C**	**C-$\hat{C}$-C**	**Egap**	**c11**	**e11**
**Bulk**	1.43	120.0	0.0	8.74	-
**monolayer**	1.42	120.0	0.0	4.37	-
**Bilayer**	1.43	120.0	0.0	8.74	-
**SW-(11,0)**	1.44	119.9	0.87	95.19	-
**SW-(20,0)**	1.43	120.0	0.46	174.05	-

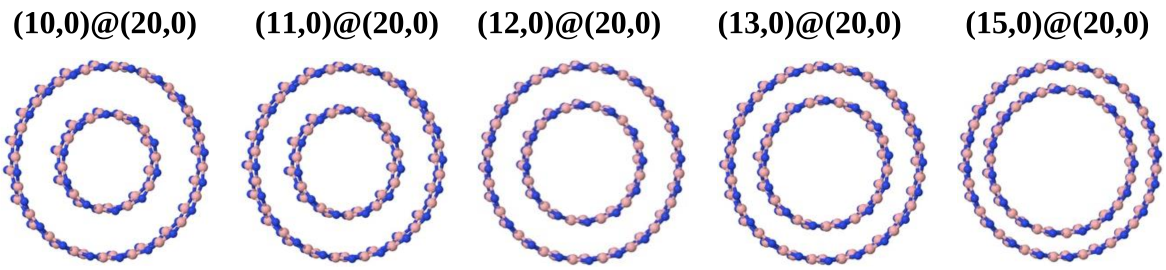

Figure S1: Unit cell of BN@BN double-walled nanotubes whose structural and energetic properties are reported in Table S2

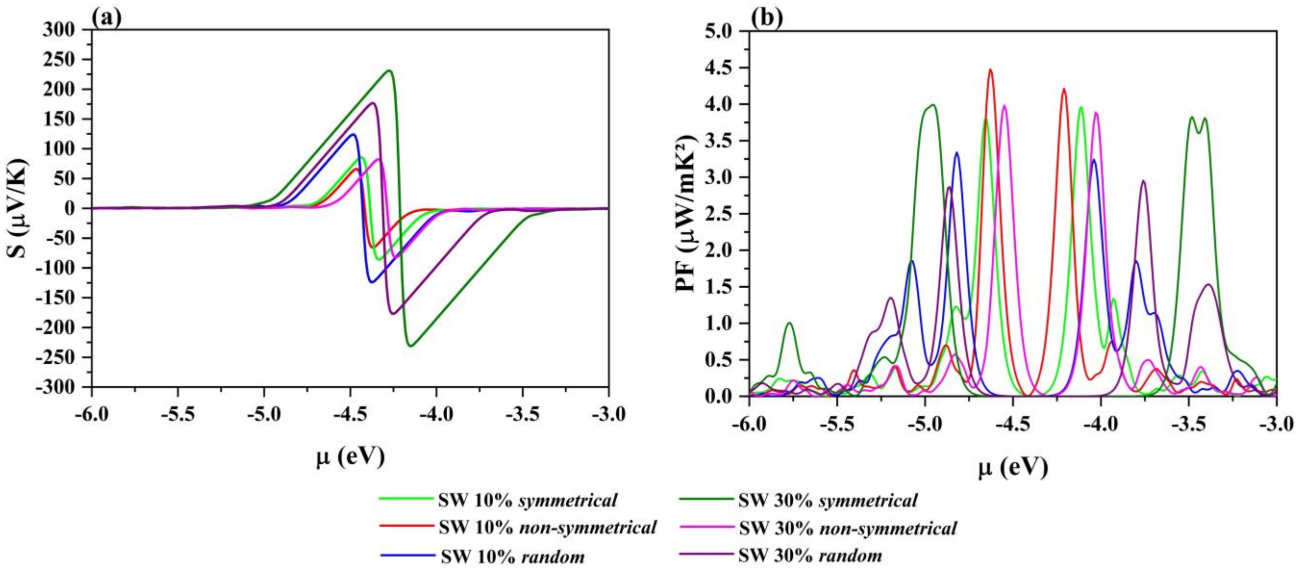

Figure S2: (a) Seebeck coefficient (μV/K) and (b) power factor (μW/mK^2) of $(BN)_{1-x}C_x$ single-wall nanotubes.

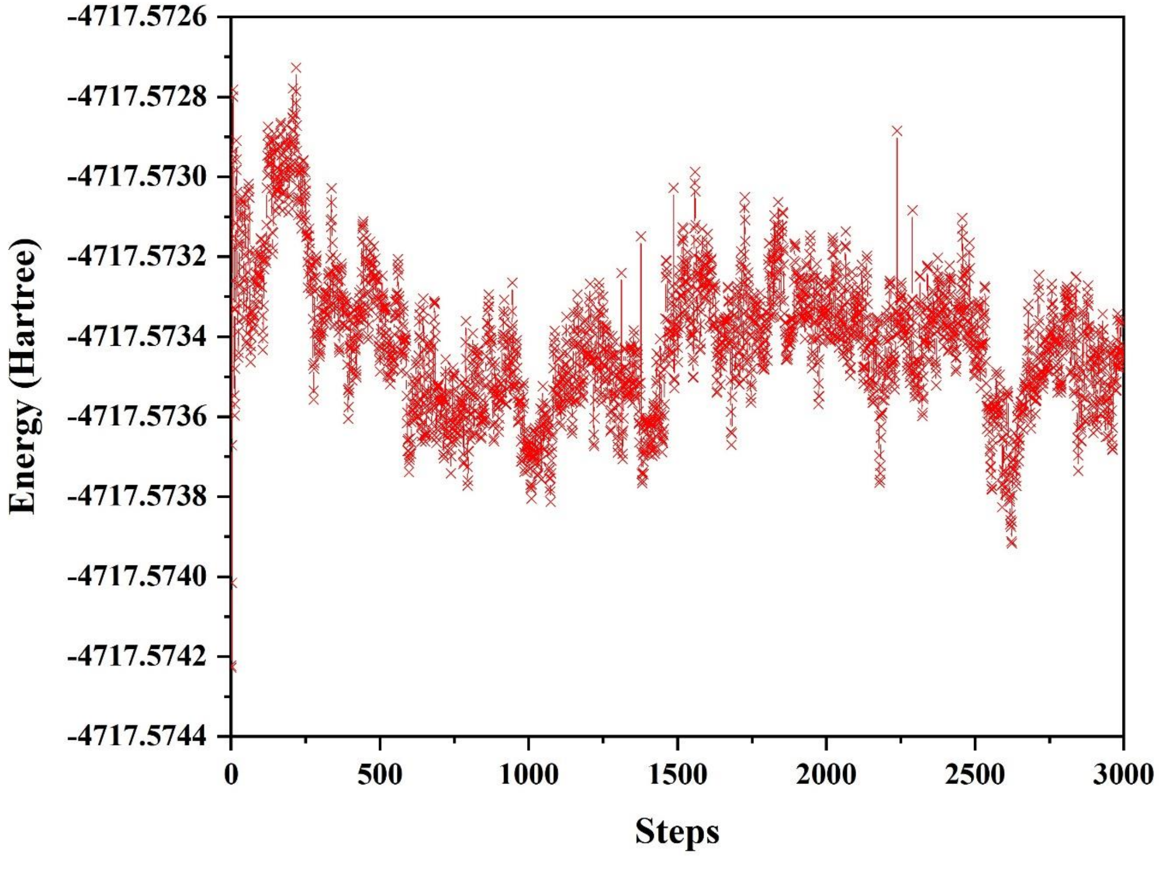

Figure S3: Molecular dynamics simulation of $C@(BN)_{0.10}C_{0.90}$ *non-symmetrical* within the canonical ensemble at T=300 K (NVT).
